# AI-Based Predictive Modelling and Alert Framework for Mortality Risk and Cost–Benefit Analysis in Rabbit Production

**DOI:** 10.3390/vetsci13040377

**Published:** 2026-04-13

**Authors:** Szilveszter Csorba, Erika Országh, Ákos Józwiák, Zoltán Német, Miklós Süth, Andrea Zentai, Zsuzsa Farkas

**Affiliations:** 1 Department of Digital Food Science, Institute of Food Chain Science, University of Veterinary Medicine, H-1078 Budapest, Hungary; csorba.szilveszter@univet.hu (S.C.); orszagh.erika@univet.hu (E.O.); jozwiak.akos@univet.hu (Á.J.); farkas.zsuzsa@univet.hu (Z.F.); 2 Department of Pathology, University of Veterinary Medicine, István u. 2, H-1078 Budapest, Hungary; nemet.zoltan@univet.hu; 3 Institute of Food Chain Science, University of Veterinary Medicine, István u. 2, H-1078 Budapest, Hungary; suth.miklos@univet.hu

**Keywords:** mortality prediction, precision livestock farming, XGBoost, threshold optimisation, cost–benefit analysis, decision support

## Abstract

Mortality events in commercial rabbit production can lead to significant economic losses, highlighting the need for earlier identification of elevated mortality risk at the group level using routinely collected production data. This study presents a machine learning–based framework for predicting mortality risk at future observation points using routinely collected production data. Models were developed using group-level variables and evaluated with StratifiedGroupKFold cross-validation to prevent information leakage. The selected XGBoost model achieved a balanced performance, with a recall of 0.78 ± 0.03, precision of 0.59 ± 0.04, and ROC–AUC of 0.72 ± 0.02. Predictions were translated into an alert system based on a predefined threshold, prioritising sensitivity while maintaining a moderate false alert rate. A scenario-based cost–benefit analysis indicated that economic outcomes are highly dependent on intervention effectiveness, with positive returns observed under moderate to optimistic assumptions. Overall, the framework demonstrates the feasibility of integrating predictive modelling with alert-based decision support in rabbit production, although real-world validation under commercial farm conditions is required to confirm its practical effectiveness.

## Simple Summary

Rabbit production systems are vulnerable to sudden increases in mortality, which can lead to significant economic losses. Early identification of elevated risk at the group level could support more timely management responses. In this study, we developed a machine learning–based framework to predict mortality risk at future observation points using routinely collected farm data. The system translates predictions into alerts that can assist decision-making at the production level. In addition, a scenario-based economic analysis was conducted to evaluate the potential financial implications of the approach. The results indicate that the framework can support earlier identification of high-risk situations, although its practical value depends on the effectiveness of management interventions following alerts.

## 1. Introduction

One of the challenges of sustainable agriculture is producing more food for a rapidly growing population, which puts pressure on crop and livestock production, contributing to environmental challenges. On the other hand, smart farming technologies are becoming increasingly prevalent in modern agriculture, promoting the optimisation of crop and livestock production while minimising waste and costs [1]. Studies have demonstrated the use of advanced data analytical (e.g., machine learning (ML)) techniques for non-contact phenotyping, performance estimation, and animal identification, even under limited data availability [2,3].

Advanced machine learning tools are emerging in animal production to monitor health and predict adverse events. For example, deep learning and tree-based models have been used in dairy and beef cattle to detect diseases (e.g., mastitis or bovine respiratory disease) and forecast morbidity or mortality with high accuracy [4]. In poultry, time-series feeding and behaviour data have enabled ML models to predict impending mortalities in broiler flocks 1–3 days in advance [5], while in intensive swine systems, ML algorithms trained on production and environmental features have produced daily outbreak risk estimates that support early intervention [6]. Precision technologies in rabbit farming are still emerging and have been identified as especially valuable for early disease detection and welfare monitoring [7]. In one recent review, early signs of digestive disorders in group-housed growing rabbits were highlighted as a high-potential target for automatic detection [7]. Overall, existing studies across livestock species—including cattle, pigs, poultry, and (more recently) rabbits—demonstrate that ML methods can forecast health problems, detect animals at risk, and ultimately improve management decisions.

Daily weight gain and feeding patterns are well-known indicators of animal health status. Animals that become ill or stressed typically eat less and gain weight more slowly, often in the days before severe symptoms or death manifest. Evidence across species supports this relationship: in pigs, feeding behaviour “represents the welfare and health status of pigs” and substantial drops in feed or water intake (e.g., 20–30%) can signal disease outbreaks like swine influenza [8]. while in rabbits, low early growth and poor feed intake have been linked to higher post-weaning mortality [9]. Similarly, time-series features derived from feeding activity have been identified as strong predictors of mortality risk in poultry systems [5]. Taken together, these findings provide the rationale for using deviations in weight gain and feeding behaviour as “early-warning” signs. Overall short-term forecasts act as a rolling “heat map” of risk: they catch emerging problems before they grow into herd-wide losses. Despite these advances, a critical gap remains in rabbit production systems: current studies largely lack operational predictive tools that are directly linked to management decisions and validated under commercial farming conditions. Recent literature further emphasises that environmental and management-related factors—including housing conditions and stress exposure—substantially influence performance and health outcomes [10], which highlights the need for predictive frameworks that not only detect risk patterns, but also integrate these context-specific factors and translate predictions into actionable decision support at the production level.

Predicting mortality has direct economic value because each saved animal represents avoided loss of feed, labour, and breeding investment. Fattening rabbits have significant input costs (feed, housing, labour) per animal, so reducing deaths translates to higher production efficiency. Formal cost–benefit analyses in other species support this: for example, Heinen et al. (2024) evaluated ML models for predicting feedlot cattle deaths and found that operating at high specificity (>90%) could yield positive net returns compared to current practices [11]. Likewise, simulations of sensor-assisted health monitoring in dairy cows (rumen bolus systems) estimated net returns of roughly +€23 to +€119 per cow-year in herds with poor baseline health [12]. These gains came from earlier disease detection leading to milder illness outcomes, reduced treatments, and less production loss. Consequently, a mortality alert framework can be used in a cost–benefit framework: model alerts trigger interventions that prevent some proportion of predicted deaths. The benefits can be quantified as the number of rabbits “saved” multiplied by their market value (minus any treatment costs). In this way, even moderate improvements in early detection can easily offset the cost of the monitoring system through reduced mortality and improved feed conversion.

However, in rabbit production, the economic dimension of predictive mortality models remains largely unexplored, especially in connection with operational decision-making and intervention strategies. This represents a critical gap between methodological development and practical implementation.

To address these gaps, this study aims to develop a machine learning–based predictive alert framework to forecast mortality risk at future observation points (approximately 2–3 weeks depending on observation intervals) for rabbit production groups, and to evaluate its economic viability through a cost–benefit analysis. Unlike daily time-series models reported in other species, our dataset is based on irregular observation intervals; therefore, a prediction horizon of 1–3 observation steps corresponds approximately to 2–3 weeks in real time.

The framework seeks to enable proactive interventions that reduce mortality losses, with the economic impact quantified by the number of rabbits potentially saved under various alert scenarios, calculated using real production values including the market price of live rabbits per kilogram and associated input costs.

## 2. Materials and Methods

### 2.1. Data Analytical Workflow

**Figure 1:**
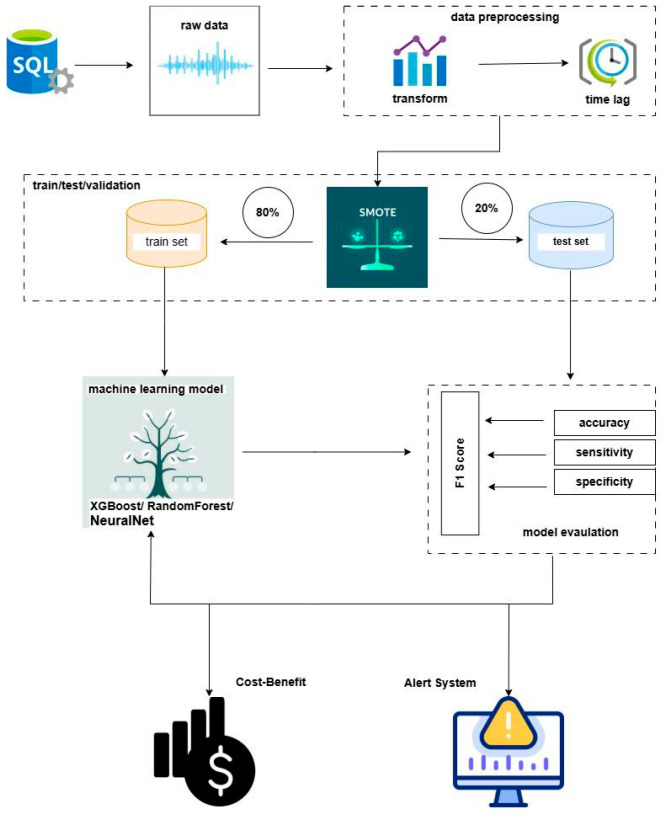
Workflow of the machine learning-based predictive modelling process: Raw data undergoes preprocessing and splitting; models are trained and evaluated on performance metrics; the final stage assesses cost–benefit and alarm system integration.

The research followed a structured machine learning pipeline for developing and evaluating the predictive model, as illustrated in Figure 1. The process began with raw data collection, followed by splitting the data into training, testing, and validation sets. The training set underwent preprocessing and SMOTE (Synthetic Minority Oversampling Technique) [13] to address class imbalance. Multiple machine learning models were trained and evaluated on the test set using performance metrics. The final stage involved a cost–benefit analysis and assessment of integration into an alarm system.

### 2.2. Data Collection and Preprocessing

Data were collected from 11 commercial fattening rabbit farms in Hungary between November 2020 and December 2025. The initial dataset comprised 5268 observations corresponding to 791 distinct production groups. Each group represents a production cohort (rotation) managed as a unit throughout the fattening cycle. Each observation corresponds to a measurement time point within a production cycle rather than strictly consecutive daily records.

To ensure robust model development, a data selection strategy was applied at the group level. Groups with insufficient observations (n < 8) were excluded to reduce instability associated with small sample sizes and limited temporal information.

As a result, the final dataset used for model training consisted of 123 production groups and 1664 observations.

### 2.3. Variables and Feature Construction

The following variables were included in the analysis:

• Group ID: Unique identifier of each production cohort, used exclusively for grouping during cross-validation to prevent data leakage. Although Group ID is used for identification and reporting within the alert system, it is not used as an input feature in model training.
• Age: Chronological age of animals in days, calculated based on controlled breeding schedules.
• Weight gain (g): Change in average group body weight between consecutive observation time points.
• Deviation from optimal weight (Diff. from opt._weight, g): Difference between observed group average weight and the expected weight derived from breed-specific production standards.
• Time interval between observations (Diff. age): Number of days between consecutive measurement time points, included to account for irregular sampling intervals.

The target variable was defined as the group-level mortality rate at each observation time point, calculated as the proportion of animals that died relative to the number of animals present in the group at that time.

All predictor variables were constructed using information available at the time of observation. No future information was used in feature construction, ensuring a strictly forward-looking modelling framework.

### 2.4. Model Development and Validation

The predictive task was formulated as a binary classification problem, distinguishing between acceptable and non-acceptable mortality risk at future observation time points.

This formulation was chosen to align the modelling task with its intended practical application. In a farm-management context, the primary decision is whether a production group requires additional attention or intervention (alert) or not (no alert), rather than assigning multiple mortality-risk categories. Therefore, the binary approach was considered more operationally relevant and easier to interpret than a multiclass formulation.

Models were trained to predict mortality risk at subsequent observation points (lag = 1, 2, 3) using predictor variables available at the current time step. The final model configuration (lag = 3) was selected based on empirical performance, providing the best balance between predictive accuracy and practical applicability. To enable forward-looking prediction, the mortality rate was shifted by 1–3 observation time points (lag = 1–3), such that the model predicts future mortality outcomes based exclusively on information available at the current observation. No lagged mortality variables were used as predictors.

Among the evaluated algorithms, gradient boosting methods performed best; therefore, the final model was implemented using Extreme Gradient Boosting (XGBoost), a tree-based ensemble method well suited for structured tabular data [14].

Missing values were handled using mean imputation, applied separately within each training fold to prevent data leakage. No global imputation was performed prior to data splitting. Feature scaling was not applied, as tree-based models are invariant to monotonic transformations.

To address class imbalance, the Synthetic Minority Over-sampling Technique (SMOTE) was applied exclusively within the training folds and only to numerical predictor variables. The group identifier was not included as a predictor and was therefore excluded from the oversampling procedure. In addition, class imbalance was further handled using class weighting (scale_pos_weight), calculated dynamically based on the class distribution within each training fold. Categorical variables were not included in the oversampling procedure.

To account for the repeated-measure structure of the dataset and avoid information leakage, the model evaluation was performed using StratifiedGroupKFold cross-validation, as implemented in the scikit-learn library, which enables simultaneous preservation of class distribution and grouping structure during data splitting, thereby preventing information leakage across repeated measurements (scikit-learn v1.8) [15]. This ensured that observations from the same group were not split across training and validation sets while maintaining class balance across folds.

Model performance was estimated using nested cross-validation, with an outer loop (5 folds) for performance estimation and an inner loop for model selection and threshold optimisation.

Instead of applying a fixed classification threshold, a data-driven threshold optimisation procedure was used. Within each training fold, prediction probabilities were generated using inner cross-validation, and candidate thresholds (0.20–0.80) were evaluated based on F1-score, precision, recall, and accuracy. A classification threshold of 0.20 was applied to convert predicted probabilities into binary outcomes. Observations with predicted probability ≥ 0.20 were classified as non-acceptable (alert), while those with probabilities below this threshold were considered acceptable (no alert). To ensure practical applicability, a minimum precision constraint was imposed. Among eligible thresholds, the final threshold was selected by maximising F1-score, with secondary consideration given to balanced accuracy and recall.

Model performance was evaluated using complementary metrics, including accuracy, balanced accuracy, precision, recall, F1-score, ROC-AUC, and average precision (PR-AUC). Metrics were calculated for each outer fold and reported as mean ± standard deviation.

In addition to standard performance metrics, a cost-sensitive evaluation framework was applied to reflect the asymmetric impact of prediction errors. False positive and false negative outcomes were assigned different penalty weights, and multiple cost scenarios were evaluated to assess robustness under varying assumptions. This analysis supports threshold selection under practical deployment conditions.

Importance values were calculated for each cross-validation fold and summarised as mean and standard deviation.

This approach is particularly suitable for longitudinal or grouped data structures, where standard random cross-validation may lead to optimistic bias due to leakage across correlated observations.

### 2.5. Alert Framework Design and Workflow

The predictive model was integrated into an alert framework designed to support early identification of elevated mortality risk at the group level.

The system operates on a time-point–based prediction horizon, where alerts are generated for mortality risk at future observation points (lag = 3). Given the irregular measurement intervals in the dataset, this corresponds approximately to a 2–3 week early warning window, providing sufficient time for pre-emptive management interventions.

The alert mechanism is based on the binary classification output of the model. An alert is triggered when the predicted probability exceeds the optimised classification threshold corresponding to the non-acceptable category (≥0.20). Predictions below this threshold are not escalated, thereby reducing unnecessary alerts.

For each triggered alert, the system generates a structured notification containing:

• the production group identifier,
• the future observation time point at which elevated mortality risk is predicted, and
• the assigned risk category (acceptable vs. non-acceptable).

This framework translates model predictions into actionable information for farm management, enabling targeted, group-level interventions.

The performance of the alert system was evaluated using standard classification metrics, including precision, recall, and F1-score, complemented by confusion matrix–based analysis to assess both detection capability and false alert rates under the operational threshold.

Importantly, the alert system is designed as a decision-support tool. Predictions indicate elevated risk rather than deterministic outcomes and should therefore be interpreted as signals prompting further inspection rather than guaranteed forecasts of mortality events.

In practice, alerts are intended to trigger targeted inspection of the affected production group, followed by context-dependent management decisions if an emerging problem is confirmed. Typical responses include closer monitoring of animal condition, evaluation of feeding and drinking systems, adjustment of feed composition, and basic veterinary assessment.

The study assumes low-cost, routine management interventions rather than intensive treatment protocols. Intervention outcomes are not explicitly modelled; instead, intervention effectiveness is incorporated as a scenario parameter in the economic analysis.

### 2.6. Cost–Benefit Analysis Framework

The economic impact of the predictive system was evaluated using a scenario-based cost–benefit analysis (CBA) framework. The analysis was directly linked to model predictions through the confusion matrix structure. True positives (TP) represent correctly identified high-risk events and corresponding opportunities for loss mitigation. False positives (FP) represent unnecessary alerts that lead to intervention costs. False negatives (FN) correspond to missed high-risk events and unrealised mitigation opportunities. True negatives (TN) have no associated cost or benefit.For each observation, the future mortality burden was defined as the number of animals that died in subsequent observation points within the same production group following the prediction time, ensuring temporal alignment between predictions and observed outcomes.

Benefits were derived exclusively from true positive predictions and calculated based on the number of animals potentially saved through intervention. Saved animals were defined as:

Saved animals = Σ (future mortality burden × intervention effectiveness)

where intervention effectiveness represents the proportion of future mortality that could potentially be prevented following an alert.

Total economic benefit was calculated as:

Total benefit (€) = saved animals × unit value per animal

The unit value per animal was assumed to be approximately 10 EUR, representing an approximate net production value at slaughter rather than gross market price.

Costs were associated with alert-triggered interventions and were applied to all alerts (TP + FP). Total cost was calculated as:

Total cost (€) = number of alerts × intervention cost per alert

The intervention cost per alert was assumed to be approximately 2 EUR and represents low-intensity management actions such as additional inspection, minor feeding adjustments, veterinary assessment, or simple group-level management changes. These costs do not include intensive veterinary treatment or other high-variability expenses.

Economic performance was quantified using the following metrics:

Net benefit (€) = total benefit − total cost

Benefit–cost ratio (BCR) = total benefit/total cost

Due to the absence of empirical intervention outcome data, multiple scenarios were evaluated using different assumptions for intervention effectiveness (e.g., 10%, 20%, 30%), allowing assessment of economic robustness under varying levels of intervention success.

This simplified formulation avoids double counting by treating animal value as a net outcome and intervention costs as aggregated operational expenses. The analysis intentionally excludes high-variability cost components (e.g., medication, labour, and diagnostics), focusing instead on a generalisable and comparable cost structure.

The results of the CBA should therefore be interpreted as an indicative assessment of potential economic impact rather than a precise financial forecast, as intervention effectiveness was not empirically validated.

The analysis focuses on incremental production-level impacts rather than large-scale outbreak prevention, reflecting the intended use of the system for routine risk management.

Because no empirical data were available on actual farmer responses or on the effectiveness of alert-triggered interventions, the economic analysis should be interpreted strictly as a scenario-based simulation. It does not demonstrate realised economic outcomes under field conditions, but rather illustrates the potential range of outcomes under different assumptions.

All analyses were performed in Python (version 3.10) using the scikit-learn (version 1.2.2), XGBoost (version 1.7), and imbalanced-learn (version 0.10) libraries. Data processing and model development were conducted in a reproducible computational environment.

## 3. Results

### 3.1. Exploration of Historical Data

**Figure 2:**
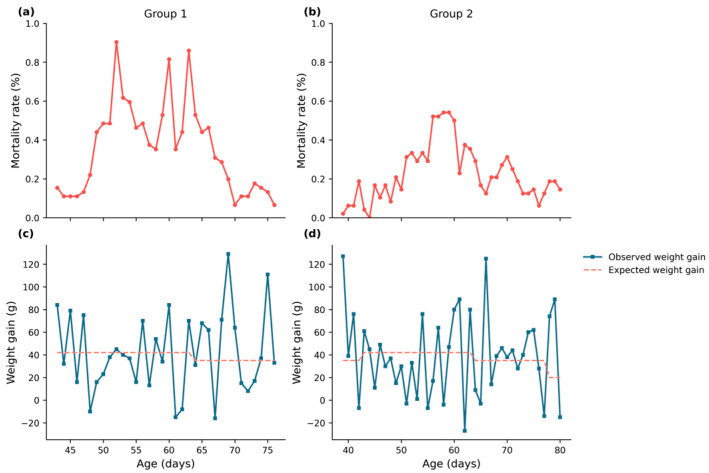
Temporal patterns of mortality and weight gain in two representative production groups. Panels (**a**,**b**) show mortality rate (%), while panels (**c**,**d**) present observed daily body weight gain (solid line) and expected weight gain (dashed line) as a function of age (days). In both groups (**a**,**c**) and (**b**,**d**), periods of reduced weight gain appear to be followed by increases in mortality after a delay. These examples illustrate a temporal association that motivated the development of medium-term predictive models.

Before model construction, an exploratory graphical analysis was performed to examine the temporal relationship between growth performance and mortality. Figure 2 presents two representative production groups. Figure 2, (a) and (b) show the mortality rate (%), while (c) and (d) display daily body weight gain (g) together with the expected growth benchmark. Visual inspection suggests a recurring temporal pattern in which periods of declining weight gain tend to precede increases in mortality. In the first group (a), (c) a marked reduction in weight gain is observed around day 48, followed by a period of sustained low or negative growth. Subsequently, mortality increases after approximately day 52 (a). A similar pattern is observed between days 55 and 65, where a prolonged reduction in weight gain (c) is followed by a corresponding increase in mortality (a). A comparable sequence is visible in the second group (b), (d) where a period of reduced or negative weight gain before day 48 (d) is followed by an increase in mortality after day 51 (b).

While these observations do not imply a causal relationship, they indicate a consistent temporal association between growth performance and subsequent mortality, which may be exploited for risk prediction.

### 3.2. Model Performance Across Time-Lag

The predictive performance of the model was evaluated across multiple prediction horizons (lag = 1, 2, and 3) to assess the trade-off between predictive accuracy and practical forecasting value (Table 1). Shorter prediction horizons (lag = 1 and lag = 2) achieved higher recall values (0.84 ± 0.03 and 0.87 ± 0.03, respectively), indicating increased sensitivity in detecting high-risk events. However, this was accompanied by lower precision and specificity, reflecting a higher rate of FP alerts.

In contrast, the lag = 3 model showed a more balanced performance profile, with improved precision (0.59 ± 0.04) and specificity (0.59 ± 0.03), while maintaining acceptable recall (0.78 ± 0.03). This indicates better discrimination between acceptable and non-acceptable cases and a reduced false alarm burden.

Overall, model performance did not monotonically decrease with increasing prediction horizon, but instead reflected a shift in the balance between sensitivity and selectivity. While shorter lags favour detection of high-risk events, longer prediction horizons provide more conservative and operationally manageable alert behaviour.

Based on this trade-off, the lag = 3 configuration was selected for further analysis, as it provides the most appropriate balance between early warning capability and acceptable FP rates. The relatively low variability across cross-validation folds indicates stable model performance and consistent generalisation across groups.

The choice of lag depends on the intended application: lag 1 prioritises sensitivity, whereas lag 3 provides a more balanced trade-off between sensitivity and specificity.

**Figure 3:**
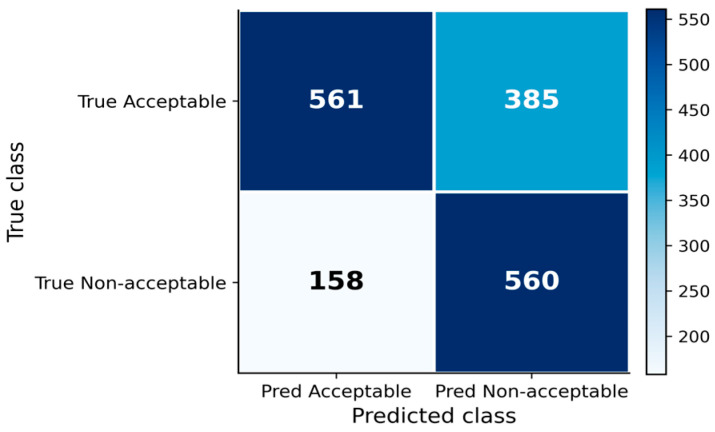
Confusion matrix of the binary classification model (lag = 3) under the selected decision threshold (0.20). The matrix summarises the distribution of TP, FP, TN, and FN predictions. The results illustrate the model’s ability to detect non-acceptable mortality risk while maintaining a balance between sensitivity and false alert rate, forming the basis for the operational alert system.

The confusion matrix (Figure 3), calculated at the selected operational threshold (0.20), provides a detailed breakdown of classification outcomes for the lag 3 model. The model correctly identified a substantial proportion of high-risk events (TP), while also generating a moderate number of FP alerts. This pattern is consistent with the model’s sensitivity-oriented behaviour.

**Figure 4:**
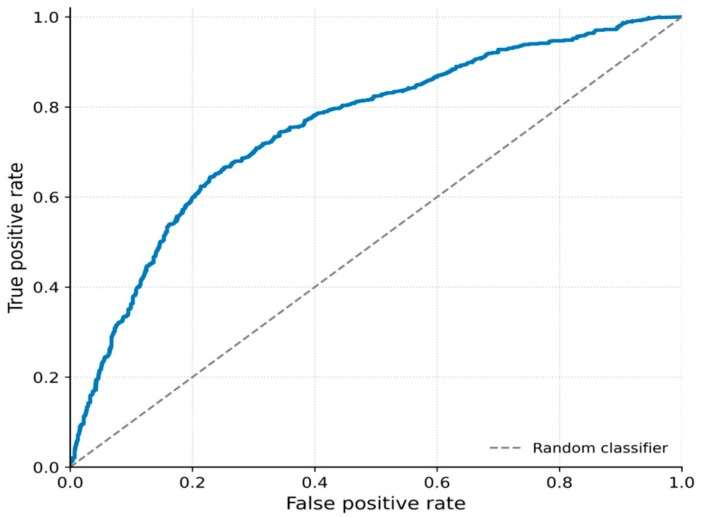
Receiver operating characteristic (ROC) curve of the lag = 3 model. The curve illustrates the trade-off between sensitivity and specificity across classification thresholds. The area under the curve (ROC–AUC = 0.72) indicates moderate discriminative performance in distinguishing between acceptable and non-acceptable mortality risk.

The receiver operating characteristic (ROC) curve (Figure 4) demonstrates that the model has acceptable discriminative ability, indicating that it can distinguish between acceptable and non-acceptable risk states better than random classification, although without high predictive certainty.

**Figure 5:**
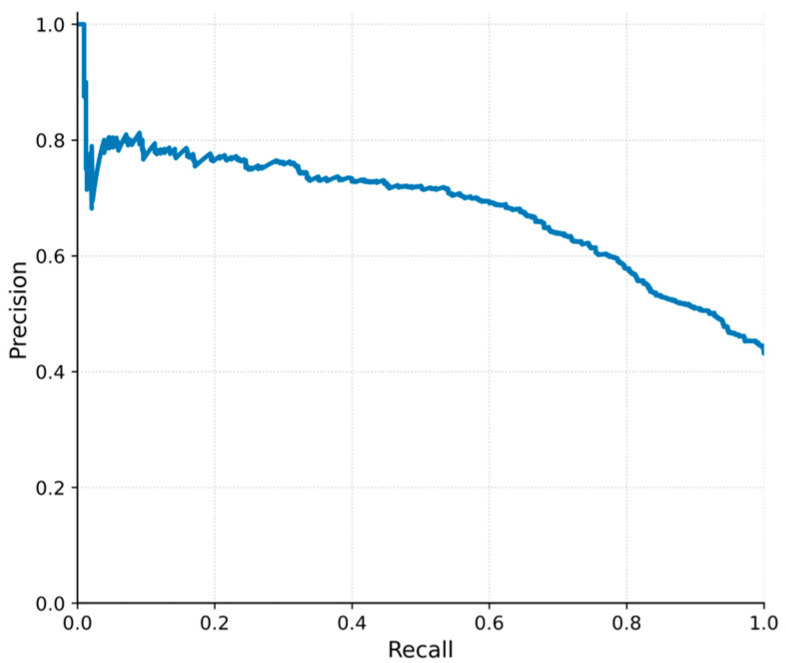
Precision–recall curve of the lag = 3 model. The curve demonstrates the trade-off between detection sensitivity (recall) and the reliability of alerts (precision) across classification thresholds. Higher recall values are associated with a gradual decline in precision, indicating an increasing proportion of FP alerts.

The precision–recall (PR) curve (Figure 5) provides additional insight into model performance under class imbalance. As recall increases, precision declines, indicating that improving sensitivity in detecting high-risk events is associated with an increasing number of FP alerts.

Overall, the model achieved a recall of 0.78 ± 0.03, a precision of 0.59 ± 0.04, and a specificity of 0.59 ± 0.03, indicating a balanced performance with a slight emphasis on sensitivity. These results confirm that while the model prioritises sensitivity, it maintains a reasonable balance between detection capability and false alarm rate.

### 3.3. Alert System Performance

The operational performance of the alert system derived from the lag = 3 model is summarised in Table 2. Performance metrics were calculated from the confusion matrix under the applied decision threshold (0.20), where predictions were mapped to alert vs. no-alert outcomes.

The system generated alerts for 57% of observations, indicating a relatively high alert frequency. The recall of 78% demonstrates that the majority of high-risk events were successfully detected. However, the specificity 59% and precision 59% indicate a moderate rate of FP alerts, reflecting the trade-off between early detection and alert burden.

The negative predictive value of 78% suggests that non-alert predictions are relatively reliable, supporting the model’s ability to identify low-risk situations. Overall, the results indicate that the alert system prioritises sensitivity, ensuring that most high-risk events are detected, while maintaining an acceptable level of false alarms for practical application.

### 3.4. Feature Importance and Exploratory Variables

**Figure 6:**
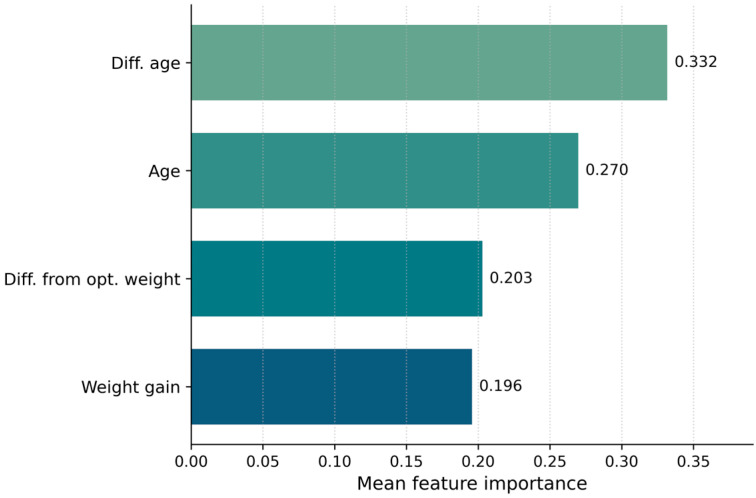
Feature importance of the final binary classification model (lag = 3). The time interval between observations (Diff. age) and Age were the most influential predictors, followed by Diff. from opt. weight and Weight gain. Values represent mean importance across cross-validation folds.

Feature importance analysis of the final binary classification model (lag = 3) indicated that Diff. age (0.332 ± 0.02) and Age (0.270 ± 0.008) were the most influential predictors, followed by Diff. from opt_weight (0.203 ± 0.009) and Weight gain (0.196 ± 0.01) (Figure 6).

The high importance of Diff. age, representing the time interval between consecutive observations, highlights that the interpretation of production-related changes is strongly dependent on the temporal structure of the data. This suggests that similar changes in growth performance may carry different predictive significance depending on the length of the observation interval.

Age was the second most important feature, reflecting the biological variability of mortality risk across different stages of the production cycle. Its contribution indicates that the model evaluates growth-related variables within an age-dependent context rather than applying uniform decision rules.

Among the growth-related variables, Diff. from opt._weight and Weight_gain showed comparable importance. Diff. from opt. weight curve captured longer-term underperformance relative to expected growth trajectories, whereas Weight gain reflected medium-term fluctuations in production dynamics.

Overall, the model relies on a combination of temporal context (Diff. age), biological stage (Age), and growth-related indicators (Diff. from opt._weight and Weight_gain) to identify observations associated with increased mortality risk.

### 3.5. Threshold Selection and Decision Trade-Off

The selection of the classification threshold plays a central role in translating model predictions into actionable decisions and their associated economic consequences. To quantify this relationship, a threshold-dependent cost analysis was performed, in which the confusion matrix was recalculated for each threshold and converted into expected economic cost per observation as described in Section 2.6.

**Figure 7:**
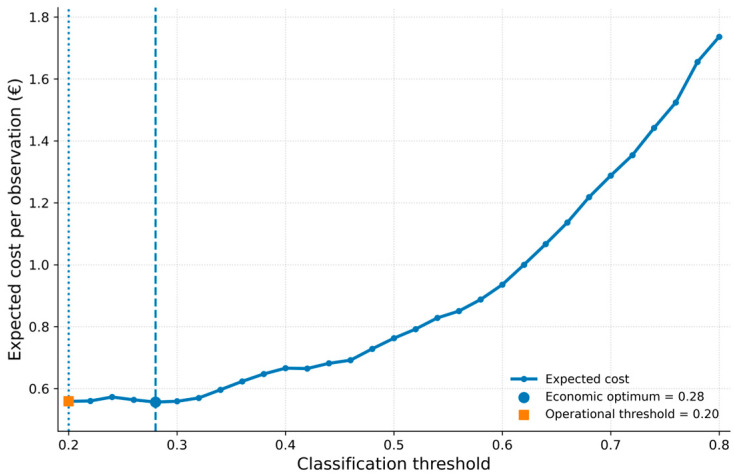
Threshold-dependent expected economic cost per observation. The expected cost was calculated based on the confusion matrix at each classification threshold, incorporating both intervention costs (FP) and losses associated with missed high-risk events (FN). The curve exhibits a minimum at approximately threshold = 0.28, representing the economically optimal operating point under the assumed cost structure. However, the cost function shows a relatively flat region around the minimum, indicating that a range of thresholds yields similar economic outcomes. The dashed vertical line indicates the economically optimal threshold, while the dotted line represents the operational threshold (0.20) used in the predictive model. The results illustrate the trade-off between increased intervention costs at lower thresholds and increasing losses at higher thresholds.

As shown in Figure 7, the expected economic cost varies systematically across threshold values. Lower thresholds result in a higher number of alerts, increasing intervention-related costs, while higher thresholds reduce unnecessary interventions but increase losses associated with missed high-risk events.

The minimum expected cost was observed at a threshold of approximately 0.28, representing the economically optimal operating point under the assumed cost structure. However, the cost function exhibits a relatively flat region around this minimum, indicating that a range of thresholds yields very similar economic outcomes.

The operational threshold (0.20) was selected based on sensitivity-oriented considerations, resulting in only a marginal increase in expected cost while substantially reducing missed high-risk events.

### 3.6. Cost–Benefit Analysis

The economic implications of the predictive model were evaluated using the cost–benefit framework described in Section 2.6. The analysis was conducted using the operational threshold (0.20), with economic outcomes calculated under different intervention-effectiveness scenarios.

Economic outcomes varied substantially across intervention-effectiveness scenarios (Table 3). Under conservative assumptions (10% effectiveness), the model resulted in a net loss (BCR = 0.77). In contrast, moderate (20%) and optimistic (30%) scenarios showed positive economic potential, with BCR values of 1.19 and 1.55, respectively. The number of saved animals increased proportionally with assumed intervention effectiveness, indicating that economic outcomes are highly sensitive to intervention success. These results indicate that the economic viability of the system is highly sensitive to intervention effectiveness. However, the analysis is based on assumed intervention success rates and simplified cost structures and does not include all potential cost elements, such as labour, veterinary treatment, or diagnostic procedures. Therefore, the results should be interpreted as indicative of potential economic value rather than as a complete financial evaluation.

## 4. Discussion

This study developed and evaluated a machine learning–based predictive framework for identifying elevated mortality risk in rabbit production systems using routinely collected group-level data. The results indicate that mortality risk can be predicted at future observation points with moderate but consistent performance, supporting the feasibility of forward-looking risk assessment in this production context.

A key finding is the trade-off between detection sensitivity and false alert burden across prediction horizons. Shorter prediction horizons (lag = 1 and 2) provided higher sensitivity but were associated with an increased number of FP alerts, which may limit their practical applicability. In contrast, the lag = 3 configuration showed a more balanced performance profile, maintaining acceptable recall while improving precision and specificity. This suggests that extending the prediction horizon can lead to more operationally manageable alert behaviour, even if some sensitivity is reduced.

The integration of model predictions into a threshold-based alert framework represents an important step toward practical application. By translating predicted probabilities into alert/no-alert decisions, the system provides interpretable outputs that can support group-level management decisions. However, the results also demonstrate that alert-based systems inherently involve a trade-off between early detection and false alarm rate. In this study, the selected threshold prioritised sensitivity, ensuring that most high-risk events were detected, while maintaining a moderate level of false alerts.

The feature importance analysis highlights the combined role of temporal structure, biological stage, and growth-related indicators in mortality risk prediction. The importance of the time interval between observations (Diff. age) suggests that the interpretation of production data depends strongly on sampling frequency, with similar changes in performance carrying different implications depending on the time span considered. Age was also a key predictor, indicating that mortality risk is not uniform across the production cycle but varies according to biological stage. Growth-related variables, including Diff. from opt. weight and Weight gain, contributed similarly to model performance, reflecting both longer-term deviations from expected development and shorter-term fluctuations in production dynamics.

These findings are consistent with previous studies in livestock systems, where reduced growth performance and altered feeding behaviour have been associated with increased disease risk and mortality. Recent developments in artificial intelligence–based monitoring in rabbit production further support the potential of such approaches for improving health surveillance and management decision-making [16]. However, it is important to emphasise that the present study identifies statistical associations rather than causal relationships. While declines in growth indicators may precede mortality events, they should be interpreted as risk signals rather than direct causes.

Several limitations should be considered when interpreting the results. First, the analysis was based on aggregated group-level data, which may obscure individual-level variability and limit the detection of early signs in individual animals. Second, although group-based cross-validation was applied to prevent information leakage, the model was trained and evaluated within a limited number of farms, and therefore its generalisability to new production environments remains uncertain. Future studies should include farm-level external validation to better assess transferability. The framework has not yet been validated under real farm operating conditions, and therefore its practical effectiveness remains to be confirmed.

In addition, the model relies exclusively on production-related variables and does not incorporate other potentially informative data sources, such as environmental conditions, feed intake, or behavioural indicators. The integration of environmental monitoring systems (e.g., temperature, humidity, and gas concentrations) using IoT-based technologies has been shown to improve livestock management and may further enhance predictive performance in rabbit production systems [17].

The economic analysis provides an indicative assessment of the potential value of the predictive framework and highlights the sensitivity of outcomes to intervention effectiveness. Under conservative assumptions, the system may not generate positive returns, while moderate to optimistic scenarios suggest potential economic benefit.

However, intervention effectiveness was not empirically validated, and several cost components, such as labour, veterinary treatments, and diagnostic procedures, were not explicitly included. Therefore, the economic results should be regarded as scenario-based estimates rather than precise financial projections.

From a practical perspective, the proposed framework should be interpreted as a decision-support tool rather than a fully validated intervention system. Predictions indicate elevated risk and may support earlier inspection and management responses, but they do not guarantee that adverse events will occur or that interventions will be effective. The actual impact of such a system depends on how alerts are interpreted and acted upon in real-world production settings. The effectiveness of such systems depends not only on predictive performance but also on the timeliness and appropriateness of the management response following each alert.

It is important to note that the economic evaluation presented in this study reflects incremental, small-scale efficiency gains at the production level rather than the prevention of large-scale disease outbreaks. The framework is designed to support earlier identification of elevated risk within individual production groups, enabling localised management responses. As such, the estimated benefits should be interpreted as arising from incremental reductions in routine mortality rather than from the avoidance of rare, high-impact epidemic events.

These simplified economic assumptions provide a transparent link between predictive performance and potential management outcomes.

Overall, the results demonstrate the feasibility of combining machine learning–based prediction, alert generation, and economic evaluation within a unified framework for rabbit production systems. While the current model provides a proof-of-concept for forward-looking mortality risk assessment, further work is required to validate its performance under real farm conditions, refine intervention strategies, and improve integration with additional data sources.

## 5. Conclusions

This study developed a machine learning–based framework for predicting mortality risk at future observation points in rabbit production systems using routinely collected group-level data. The results demonstrate that such data can be used to identify elevated mortality risk in advance, with moderate and consistent predictive performance across cross-validation folds.

The integration of predictive modelling with a threshold-based alert framework enables the translation of model outputs into actionable signals at the production level. However, the results also highlight the inherent trade-off between detection sensitivity and false alert burden, indicating that threshold selection should be considered a context-dependent decision rather than a fixed model parameter.

The economic evaluation suggests that the potential value of the system is highly dependent on the effectiveness of management responses following alerts. Under certain assumptions, the framework may support economically beneficial decision-making, but these findings are based on simplified scenarios and should be interpreted as indicative rather than conclusive. The estimated economic benefits reflect incremental improvements in production efficiency rather than large-scale disease prevention.

Overall, the proposed approach provides a proof-of-concept for integrating predictive modelling, alert generation, and economic assessment in rabbit production systems. Further work is required to validate the framework under real farm conditions, refine intervention strategies, and assess its generalisability across different production environments.

## Figures and Tables

**Table 1:** Performance of the binary classification model at different prediction horizons (lag = 1, 2, 3), reported as mean ± standard deviation across cross-validation folds.

Metric	Lag 1	Lag 2	Lag 3
Accuracy	0.70 ± 0.03	0.68 ± 0.03	0.67 ± 0.03
Balanced accuracy	0.71 ± 0.03	0.70 ± 0.03	0.69 ± 0.03
Precision	0.62 ± 0.04	0.60 ± 0.04	0.59 ± 0.04
Recall	0.72 ± 0.04	0.75 ± 0.03	0.78 ± 0.03
F1-score	0.66 ± 0.03	0.67 ± 0.03	0.67 ± 0.03
ROC–AUC	0.74 ± 0.02	0.73 ± 0.02	0.72 ± 0.02
PR–AUC	0.65 ± 0.03	0.64 ± 0.03	0.63 ± 0.03

**Table 2:** Operational performance of the alert system derived from the lag = 3 model at the selected decision threshold (0.20). Metrics were calculated from the confusion matrix under the binary alert vs. no-alert framework. The results reflect the trade-off between sensitivity and false alert burden in practical application.

Metric	Value	Interpretation
Alert rate	57%	Proportion of observations triggering an alert
Recall (sensitivity)	78%	Proportion of high-risk events correctly detected
Specificity	59%	Proportion of low-risk cases correctly identified
Precision (PPV)	59%	Proportion of alerts corresponding to true high-risk events
Negative predictive value (NPV)	78%	Proportion of non-alerts corresponding to low-risk cases

**Table 3:** Scenario-based cost–benefit outcomes under different intervention-effectiveness assumptions. Effectiveness represents the proportion of predicted high-risk cases assumed to be prevented through alert-triggered management actions.

Scenario	Intervention Effectiveness (%)	Saved Animals	Total Benefit (€)	Total Cost (€)	Net Benefit (€)	Benefit–Cost Ratio
Conservative	10	215	1720	2224	−504	0.77
Moderate	20	430	4300	3616	684	1.19
Optimistic	30	645	7740	5008	2732	1.55

## Data Availability

The original contributions presented in this study are included in the article. Further inquiries can be directed to the corresponding author.

## Abbreviations

The following abbreviations are used in this manuscript:

AI	Artificial Intelligence
AUC	Area Under the Curve
CBA	Cost–Benefit Analysis
CBR	Cost–Benefit Ratio
CI	Confidence Interval
F1 score	Harmonic Mean of Precision and Recall
FN	False Negative
FP	False Positive
IoT	Internet of Things
ML	Machine Learning
NPV	Net Present Value
PR-AUC	Area Under the Precision-Recall Curve
ROC	Receiver Operating Characteristic
SMOTE	Synthetic Minority Over-sampling Technique
TN	True Negative
TP	True Positive
XGBoost	Extreme Gradient Boosting

## References

[1] Monteiro A., Santos S., Gonçalves P. (2021). Precision Agriculture for Crop and Livestock Farming—Brief Review. Animals.

[2] Pretto A., Savio G., Gottardo F., Uccheddu F., Concheri G. (2024). A Novel Low-Cost Visual Ear Tag Based Identification System for Precision Beef Cattle Livestock Farming. Inf. Process. Agric..

[3] Xu J., Jiang W., Wang L., Wang H., Wu J., Shen Y., Zhu C., Hao S., Liu C. (2025). Recent Advances in Computer Vision for Non-Contact Phenotyping and Weight Estimation in Livestock: A Systematic Review. Inf. Process. Agric..

[4] Senthilkumar C., C S., Vadivu G., Neethirajan S. (2024). Early Detection of Lumpy Skin Disease in Cattle Using Deep Learning—A Comparative Analysis of Pretrained Models. Vet. Sci..

[5] Alves A.A.C., Fernandes A.F.A., Breen V., Hawken R., Rosa G.J.M. (2024). Monitoring Mortality Events in Floor-Raised Broilers Using Machine Learning Algorithms Trained with Feeding Behavior Time-Series Data. Comput. Electron. Agric..

[6] Halev A., Martínez-López B., Clavijo M., Gonzalez-Crespo C., Kim J., Huang C., Krantz S., Robbins R., Liu X. (2023). Infection Prediction in Swine Populations with Machine Learning. Sci. Rep..

[7] Norton T., Cambra-López M. (2025). Challenges and Opportunities for Precision Livestock Farming Applications in the Rabbit Production Sector. World Rabbit. Sci..

[8] Kim M., Choi Y., Lee J., Sa S., Cho H. (2021). A Deep Learning-Based Approach for Feeding Behavior Recognition of Weanling Pigs. J. Anim. Sci. Technol..

[9] Assan N. (2018). Factors Influencing Post-Weaning Growth and Mortality in Rabbits. Sci. J. Anim. Sci..

[10] El-Sabrout K., Sherasiya A., Ahmad S., Aggag S., Nannoni E., Cavallini D., Buonaiuto G. (2024). Environmental Enrichment in Rabbit Husbandry: Comparative Impacts on Performance and Welfare. Animals.

[11] Heinen L., White B.J., Larson R.L., Kopp D., Pendell D.L. (2024). Economic Impact of Mortality Prediction by Predictive Model at First and Second Treatment for Bovine Respiratory Disease. Am. J. Vet. Res..

[12] Pfrombeck J., Gandorfer M., Zeiler E., Ettema J. (2025). An Economic Evaluation of Sensor-Assisted Health Monitoring in Dairy Farming Using the Example of a Rumen Bolus. J. Dairy Sci..

[13] Chawla N.V., Bowyer K.W., Hall L.O., Kegelmeyer W.P. (2002). SMOTE: Synthetic Minority Over-Sampling Technique. J. Artif. Intell. Res..

[14] Chen T., Guestrin C. (2016). XGBoost: A Scalable Tree Boosting System. Proceedings of the 22nd ACM SIGKDD International Conference on Knowledge Discovery and Data Mining.

[15] Pedregosa F., Varoquaux G., Gramfort A., Michel V., Thirion B., Grisel O., Blondel M., Prettenhofer P., Weiss R., Dubourg V. (2011). Scikit-Learn: Machine Learning in Python. J. Mach. Learn. Res..

[16] Abdel-Wareth A.A.A., Ahmed A.A., Salahuddin M., Lohakare J. (2025). Application of Artificial Intelligence in Rabbit Husbandry: From Reproductive Monitoring to Precision Farming. Front. Vet. Sci..

[17] Provolo G., Brandolese C., Grotto M., Marinucci A., Fossati N., Ferrari O., Beretta E., Riva E. (2025). An Internet of Things Framework for Monitoring Environmental Conditions in Livestock Housing to Improve Animal Welfare and Assess Environmental Impact. Animals.

